# Assessment of the Occupational Health and Food Safety Risks Associated with the Traditional Slaughter and Consumption of Goats in Gauteng, South Africa

**DOI:** 10.3390/ijerph14040420

**Published:** 2017-04-14

**Authors:** Daniel N. Qekwana, Cheryl M. E. McCrindle, James W. Oguttu, Delia Grace

**Affiliations:** 1 Section Veterinary Public Health, Department of Paraclinical Sciences, Faculty of Veterinary Science, University of Pretoria, Private Bag X04, Onderstepoort, Pretoria 0110, South Africa; 2 School of Health Systems and Public Health, Faculty of Health Sciences, University of Pretoria, Pretoria 0001, South Africa; cheryl.mccrindle@gmail.com; 3 Department of Agriculture and Animal Health, College of Agriculture and Environmental Science, University of South Africa, Christiaan de Wet Rd. & Pioneer Avenue, Florida Park, Roodepoort 1710, Gauteng, South Africa; joguttu@unisa.ac.za; 4 ILRI-Kenya, Nairobi 00100, Kenya; d.grace@cgiar.org

**Keywords:** traditional slaughter, hazard identification, hygiene practices, food safety, occupational health, risk

## Abstract

Background: This study assessed the occupational health and food safety risks associated with the traditional slaughter of goats and the consumption of such meat in Tshwane, South Africa. Methods: A convenience sample of 105 respondents agreed to be interviewed using structured questionnaires. Results: A high proportion (62.64%) of practitioners admitted to not wearing protective clothing during slaughter. Slaughtering was mainly carried out by males (99%) with experience (62.2%). Forty-four percent of practitioners only changed the clothes they wore while slaughtering when they got home. During the actual slaughter, up to seven people may be involved. The majority (58.9%) of slaughters occurred early in the morning and none of the goats were stunned first. In 77.5% of cases, the health status of the persons who performed the slaughtering was not known. The majority (57.3%) of the slaughters were performed on a corrugated iron roof sheet (zinc plate). In 83.3% of the cases, the carcass was hung up to facilitate bleeding, flaying, and evisceration. Meat inspection was not practiced by any of the respondents. Throughout the slaughter process, the majority used the same knife (84.3) and 84.7% only cleaned the knife when it became soiled. A total of 52.0% of the respondents processed the carcass and cooked the meat immediately. The majority (80.0%) consumed the meat within 30 min of cooking. Conclusions: Men are at a higher risk of occupational health hazards associated with traditional slaughter, which can be transferred to their households. Unhygienic methods of processing and the lack of any form of post-mortem examination increase the risk of food-borne illness following the consumption of such meat.

## 1. Introduction

A risk-based, ”farm to fork” food safety risk assessment and mitigation approaches consider the possibility of hazards being introduced during the production, processing, and consumption of meat and meat products [1,2,3]. Certain practices at different stages of the value chain may increase or decrease the risk of food-borne and zoonotic diseases. Therefore, effective control measures must be put in place to mitigate occupational health and food safety hazards/risks throughout the value chain. Globally, there are efforts to improve and standardise food safety management systems [4,5] and a number of food safety programmes or systems, including ISO 22000, Good Hygienic Practices (GHP), and Hazard Analysis Critical Control Point system (HACCP) [6,7,8], have been developed. In South African abattoirs, the hygiene assessment system (HAS) and hazards analysis critical control points (HACCP) are used to identify hazards during the slaughter and processing of meat, so as to minimise the risk of food-borne illnesses [9].

Although the Meat Safety Act 40 of 2000 prescribes the implementation of food safety management systems in registered abattoirs, this is not the case for traditional slaughter practices [9]. Similarly, the UK [10] and the USA [11] permit the slaughter of animals for traditional or religious purposes, with minimal to no reference to food safety management systems. This is in spite of the potential for food-borne diseases in traditional slaughter being greater than in abattoir slaughter [12].

In South Africa, there is limited information on the incidence and prevalence of food-borne and zoonotic diseases associated with the traditional slaughter of goats that takes place outside of the registered abattoirs. This may be related to a lack of reporting, since most food-borne diseases are self-limiting and affected individuals do not usually seek medical attention [13,14]. Therefore, the aim of this study was to assess the occupational health and food safety risks associated with the traditional slaughter of goats and the consumption of meat in and around Tshwane.

## 2. Materials and Methods 

### 2.1. Study Area

The study was conducted among the taxi ranks (n = 4) and informal markets (n = 18) in the Tshwane Metropolitan Municipality. The site was selected because it offered access to a cross-section of people from different parts of the country, including both urban and peri-urban areas. Furthermore, at taxi ranks and informal markets, large numbers of people can be accessed in one place. Because most taxi ranks in Tshwane are located near a mall or shopping centre, it was possible to gain access to shoppers from various parts of the Gauteng Province. The 18 sites involved in this study that sold live goats were located along the roads leading to Soshanguwe, Mabopane, Brits, and Hammanskraal in the North West Province. 

### 2.2. Sampling and Study Design

A cross-sectional study design using a purposive sample was adopted for this study. A total of 300 people were approached, but only 105 agreed to be interviewed. The criteria for inclusion were that the respondents had to agree to be interviewed and must have been involved in the traditional slaughter of goats, either as a spectator or a participant. The study included both males and females from areas within and outside the city of Tshwane—both rural and urban. Trained animal health students conducted the interviews in the home language of the respondents. Consent was obtained from each participant before the interview was conducted [15,16].

### 2.3. Data Management and Analysis

The following categories of variables were included in the questionnaire: Demographic profile of the respondents; hygiene practices of the respondents; hygiene conditions during slaughtering, flaying, evisceration, and removal of the pluck; meat inspection; transportation of offal; storage and cooking of meat; and consumption of meat. Raw data were entered into Excel (Microsoft, Redmond, WA, USA). Data were evaluated for missing values and any inconsistencies before being analysed using SAS Statistical Software Version 9.4 (SAS Institute, Cary, NC, USA). Quantitative data was summarised using descriptive statistics and presented as frequency tables and graphs. Open-ended questions were analysed using the word-based technique described by Ryan and Bernard [17] (2003).

## 3. Results

### 3.1. Demographics and Hygiene Practices 

The majority (99%) of respondents performing exsanguination were males and they were selected to perform the traditional slaughter based on experience (62.22%) or because they were a family member (35.56%). Few (2%) respondents indicated that a sangoma (traditional healer) performed the exsanguination. In 50.55% of the slaughtering processes, between three and four people were involved; in 40.66% of cases, only one to two people were involved. The majority (77.53%) of respondents did not enquire about the health status of the people who carried out the slaughtering (Table 1). 

Approximately 37.36% of the respondents indicated that they do not wear protective clothing, but instead wear casual (street) clothes (97.14%) during the slaughter. Just under half (41.86%) of the respondents indicated that they changed the clothes they used during the slaughter. Of those who indicated that they changed their clothes after the slaughter, 44.12% indicated that they only changed when they get home, while 36.76% said they did so within 10 min of completing the slaughtering. The most (93.22%) commonly used protective clothes were overalls (Table 2). 

### 3.2. Slaughter Process

The majority (58.89%, 62/105) of respondents slaughter in the early hours of the morning, followed by those who slaughter in the evening (38.89%, 41/105); the minority slaughter in the afternoon (2.22%, 2/105). In 57.30% (60/105) of cases, slaughtering is performed on a corrugated iron roof sheet (zinc plate), followed by slaughter where the goat is in direct contact with the ground (39.33%, 41/105) and on a plastic bag (3.37%, 4/105). None (100%, 105/105) of the respondents indicated that stunning was performed. 

### 3.3. Exsanguination 

Respondents indicated that exsanguination starts with the dorsal extension or stretching of the neck to expose the neck area, followed by the slitting of the throat with a knife. A back and forth movement method is used to cut the skin and slit the throat, according to 98% of respondents. The rest (2%) said that they use a swift single cut. A similar observation was made during visits to four traditional slaughter ceremonies.

According to 83.3% of respondents, bleeding out occurs with the animal suspended on a tree or pole. However, in 16.87% of cases, the animal is left to bleed out while on the ground/corrugated iron sheet. The majority (74.57%) of respondents let the carcass hang for 10–20 min to bleed out. The rest (25.43%) let the carcass bleed for less than 10 min (Table 3).

### 3.4. Carcass Dressing

Two methods of flaying and dressing were described by respondents and the most common one (91.55%) starts with the carcass placed on its back with the legs facing upwards. A sharp knife is then used to make an incision starting at the medial surface of each leg from the point of the knee (carpal or tarsal joint) towards the abdomen. The cut edges of the skin are then loosened using a knife to detach the skin using the “fisting” method. The whole skin is not removed at once, but is left on the carcass to prevent the carcass from getting soiled. The feet and the head are not removed immediately; this allows them to be used to hold or lift the carcass. It is only after the skin has been completely removed that the feet and the head are cut off from the rest of the carcass.

The other method, which is not as common (8.45%), involves hanging the carcass with its hind legs tied to a pole or a tree. Then, a sharp knife is used to make an incision starting from the medial surface of each hind leg from the knee (tarsal) joint towards the abdomen. The cut edges of the skin are loosened with a knife; this may be followed by “fisting” and/or pulling of the skin downwards. As with the first method, the feet and the head are only removed when the carcass has to be moved or carried. This is the method of choice where the slaughter process is performed by fewer people (≤2).

The respondents reported washing their hands more frequently during flaying and dressing (87.91%) and, in most cases (61.25%), it involved using water without a disinfectant. In 86.06% of cases, municipal water was used, compared with borehole water in 10.13% of cases and water from rivers in 3.80% of cases. Only 12.50% of the respondents washed their knife before flaying and dressing. The largest proportion (94.38%) of respondents indicated that they used knives provided by the family; only 5.62% used their own knives (Table 3).

In both methods of slaughter described above, a small abdominal incision that fits at least two fingers is made in the abdominal cavity. This is created so that when the fingers are inserted into the abdomen, they serve as a safety measure to prevent piercing of the abdominal content. Later, the same abdominal incision is extended to expose the abdominal contents. The abdominal organs are removed before the thoracic cavity is opened up to remove its contents. The pluck is removed by making an incision through the diaphragm into the thoracic cavity to completely expose the thoracic content. At this point a strong sharp object, such as an axe, is used to cut through the sternum. When the internal thoracic cavity has been exposed, the pluck is removed and placed in a container.

Only 12.50% of respondents indicated that they put both the red and rough offals in the same container. However, the majority (87.50%) put the red and rough offals in separate containers. It is noteworthy that the respondents who indicated that they put the rough and red offals together do not consume the offal. The red offals are washed with water, cut into small pieces, and then cooked. In 18.31% of cases, rough offals are cleansed by inserting a hosepipe into the intestines or fore-stomachs and flushing the ingesta out. After the cleaning has been completed, the offals are cut into small pieces to prepare them for cooking. The other method of cleaning rough offals, which is practiced by the majority (81.69%) of respondents, involves “milking” or squeezing the intestines to remove all of the ingesta. This is accompanied by the opening up of the fore-stomachs to remove all of the ingesta.

### 3.5. Meat Inspection and Final Wash

All respondents (100%) indicated that no meat inspection was performed. However, if an abnormality is encountered during slaughtering, almost all (96.70%) of the respondents indicated that it is cut off and thrown away, buried, or gets thrown into the fire and burnt. 

### 3.6. Processing, Storage and Consumption 

After flaying and dressing have been completed, the carcass is either transported by two men who carry it by holding the legs, or by one man who carries it over his shoulder. After the carcass has been dressed, it either gets stored (in about half of the cases, 48.28%) or it gets cut up into small pieces immediately.

If the carcass is to be stored, in the majority of cases (85.71%), it is hung up by tying it to the roof in the garage or to a tree. Alternatively, it is left on a table (14.29%). Processing of the carcass involves separating the carcass at the joints and cutting the meat into small pieces. Large bones are often thrown into the fire. 

The majority of respondents (80.01%, 36/45) consumed the meat within 30 min of cooking, while the rest (19.99%, 9/45) said that they ate the meat more than 30 min after cooking. 

More than half of the respondents (55.24%, 58/105) indicated that all of the meat is consumed on the same day in one sitting, whereas 32.38% (34/105) indicated that they store leftover meat in the fridge. In terms of the duration of storage, most respondents (79.41%, 83/105) indicated that the leftover meat is stored for less than 24 h. The others (20.59%, 22/105) said that the meat gets stored for longer (more than 24 h).

## 4. Discussion

Although Newell et al. [13] argue that the present challenges of food-borne disease are similar to those observed 20 years ago, emerging and re-emerging diseases put a significant strain on the already stretched health system in many developing countries [1]. The majority of studies used to determine the burden of food-borne diseases are based on the formal food value chain, and little is known about the contribution of the informal market to this burden. Infants, children, the elderly, pregnant women, and immunocompromised individuals continue to be at a higher risk of food-borne illness [18,19]. This study describes risks associated with the traditional slaughter and consumption of goats in Tshwane, South Africa, and the occupational health risks associated with traditional slaughter practices. The present study was motivated by the need for information to guide food safety policy development for the traditional slaughter of goats.

### 4.1. Occupational Health Risk

In this study, only men were involved in the slaughter of goats. Khalili et al. [20] also observed that in Iran, only men slaughter animals. According to Battelli [21], people such as veterinarians, meat inspectors, and meat examiners are at a higher risk of occupational and zoonotic diseases [21]. Based on this, men are at a greater risk of occupational health hazards associated with the traditional slaughter of animals than their female counterparts. However, the situation is different in Jamaica, where Brown et al. [22] reported that both males (67.3%) and females (36.7%) were involved in traditional slaughter. Furthermore, since up to seven people were reported to be involved during traditional slaughter, the risk of exposure to occupational health hazards is multiplied in the community. This risk may be compounded by the limited knowledge of zoonotic diseases in communities that practice traditional slaughter, the limited studies on the prevalence of zoonotic diseases, and the limited access to veterinary services in South Africa [15]. 

In the present study, a high proportion (62.64%) of practitioners admitted to not wearing protective clothing during slaughter. This a much higher proportion than was observed among abattoir workers in Ethiopia, where Haileselassie et al. [23] reported that only 14.6% did not wear protective clothing, and in South Africa, where Nel et al. [24] reported that all (100%) abattoir workers wore protective clothing. The reason for the low proportion of people using protective clothing during traditional slaughter may be the lack of knowledge of occupational health hazards among the traditional slaughter practitioners. This is also exacerbated by the lack of formal training for traditional slaughter practitioners [16]. Traditional slaughter practitioners depend on the knowledge that is passed on informally, from one generation to the next. 

The high number of practitioners who indicated that they only changed their clothes worn during slaughtering when they got home has serious public health implications. Protective clothing protects against occupational health hazards and prevents the contamination of meat in the abattoirs [25]. Therefore, going home with clothes worn during the slaughter of animals, as was observed in this study, has the potential to increase the risk of the transmission of zoonotic and food-borne pathogens from the slaughter place to the household of slaughterers. To mitigate this risk, the authors suggest that traditional slaughterers be advised to adopt the use of disposable protective clothes during slaughter. 

### 4.2. Food Safety Risks

With regard to the health status of traditional slaughter, our findings are in agreement with the study by Nel et al. [24], who observed that the health status of the majority of workers in the deboning room in a formal abattoir was unknown. A medical examination in many countries is part of the food safety management system and it is therefore mandatory for personnel who handle food to obtain a medical health certificate. In South Africa, the Health Act 63 of 1977 states that no person may be in contact with food if such a person is suspected of suffering from or being a carrier of a disease or condition in its contagious stage that can be transmitted by food [26]. This is because meat handlers who carry an infectious disease may contaminate meat and meat products, thus increasing the risk of food-borne disease outbreaks. 

We observed that the slaughter process usually takes place in the early hours of the morning or late in the evening. During these times, the ambient temperature tends to be lower/cooler than later in the day [27]. This has the potential to minimise bacterial growth on the carcass and to increase the shelf life of the meat. This practice should be considered as a risk mitigation strategy and should be encouraged to enhance the safety of the meat. 

In this study, exsanguination and flaying were predominantly performed on a sheet of corrugated iron. The use of corrugated iron sheeting has been reported to reduce the likelihood of contamination of the carcass and has been encouraged by reputable organisations such as the FAO [28]. In addition, corrugated iron sheeting prevents the exposure of the carcass to environmental hazards and unhygienic surfaces, and thus prevents or minimises the risk of food-borne pathogens [29].

Blood is an ideal medium for bacterial growth, and therefore, improper bleeding may result in a reduction in the quality and shelf life of the meat [30]. In this study, it was observed that in the majority of cases during traditional slaughter, carcasses were hung for more than the recommended bleeding time of six minutes [9]. This practice should be encouraged because it has the potential to enhance both the safety and shelf life of the meat from animals that are traditionally slaughtered. In view of this, the authors recommend that during traditional slaughter, the practice of hanging carcasses to allow for adequate bleeding should be encouraged [8,9] because of its potential to reduce the likelihood of food-borne disease or illnesses [28,31]. In addition, a carcass in a hanging position ensures the minimum risk of exposure to environmental hazards and facilitates a smooth flaying and dressing process. Various cost-effective methods, such as trees and poles, which have been suggested by the FAO [28], can be adopted. 

Although the majority of respondents washed their hands before flaying, the utensils used, such as knives, were not washed regularly during flaying. Dirty knives are a potential source of contamination and inadequately cleaned equipment has been linked to carcass contamination during the slaughter and outbreaks of food-borne diseases [32]. However, it was encouraging to note that the majority of respondents used municipal water, which is the most potable source of water in South Africa. 

The study found that the flaying process in traditional slaughter practices was similar to that of formal slaughter in the abattoir, with few exceptions [9]. One exception is that in abattoirs, machines are used to remove the skin, whereas in traditional slaughter, the skin is removed by “fisting”. The fisting process involves direct contact being made between the hands and the carcass, which is a potential source of carcass contamination. Since the water used to wash the hands during the traditional slaughter does not include a disinfectant, a risk mitigation strategy needs to be implemented at this stage. This could take the form of the traditional slaughterers’ using a disinfectant in the water that they use to wash their hands and utensils, provided it does not leave a chemical residue in the meat. 

The rupture of the viscera contents may result in the contamination of the carcass. Therefore, every effort must be made to ensure that they are removed intact [33]. It was clearly evident that the traditional slaughter practitioners are fully aware of this fact. Therefore, they have adopted the method of using two fingers inserted into the abdominal cavity to avoid puncturing the abdominal content during evisceration. However, the act of inserting two fingers into the small incision made in the abdomen could act as a source of contamination. In view of this, traditional slaughter practitioners must wash their hands thoroughly to minimise the contamination of the carcass. As in the case of formal slaughter in the abattoir, during traditional slaughter, only the inside of the carcass is washed with water after evisceration and it is then hung up to dry. This is consistent with the requirements of the Meat Safety Act 40 of 2000 [9]. 

The transportation of red and rough offals in separate containers is an important food safety mitigation step that must be encouraged among traditional slaughter practitioners. In this study, the respondents did not seem to appreciate the importance of separating the two categories of offal. Moreover, during transportation, the carcass should be covered with plastic material to prevent direct contact between the clothes of those carrying the carcass and the meat. This will help to reduce the risk of carcass contamination during transportation. Furthermore, in cases where meat is stored overnight, it should be kept in a place or an area that does not increase the likelihood of contamination of the carcass, namely a dry, clean, cool place, where the carcass is elevated off the ground. 

The role of a meat inspection service in safeguarding or assuring food safety and quality has been well documented in the literature [34]. In this study, it was noteworthy that no primary or secondary meat inspection was reported to have been performed. Although the authors are aware of the challenges of implementing a meat inspection service in traditional or informal slaughter, a simplified community-based programme of food safety could be developed for traditional slaughter practitioners. In addition, a semi-formal training qualification similar to that of meat examiners could be developed to bridge the gap between traditional slaughter and the formal slaughter that takes place in the abattoir. In the absence of information on goat diseases in South Africa, meat inspection plays an important role in reducing the risk of food-borne illness among traditional slaughter practitioners and those who consume such meat. 

The study showed that meat derived from a ritual slaughter was often consumed immediately, with few exceptions. This confirmed the findings of Gchabashe [35], who reported that meat from traditional ceremonies is consumed immediately after slaughter. Together with long periods of cooking, this practice is important in mitigating the risk of food-borne illnesses during traditional slaughter ceremonies [36]. 

### 4.3. Limitation of the Study 

The limitation of this study was that the study was limited to people who make use of taxi ranks in different parts of the city of Tshwane and/or those who practice the traditional slaughter of goats. Therefore, practitioners who do not make use of taxi ranks or source goats from the informal markets were not included in the study. Consequently, the results of this study must be interpreted without casting general conclusions for the entire country. Nonetheless, this study provides baseline information on the occupational health and food safety risks associated with the traditional slaughter of goats. 

## 5. Conclusions 

Although both genders are involved in the traditional slaughter of goats, it is males who are at a higher risk of exposure to occupational health hazards by virtue of the fact that the actual slaughtering is mainly performed by men. This risk can be transferred to family members of the traditional slaughter practitioners and the general public because of the practice of slaughtering while wearing street clothes. Traditional slaughter practitioners have developed some risk mitigation strategies with the potential to limit both occupational health and food-borne illness; therefore, these strategies should be promoted. However, poor hygiene practices while processing the meat and the lack of any form of post-mortem examination are issues that increase the risk of food-borne illness. Therefore, there is a need to develop food safety management programmes for traditional slaughter to mitigate the risk associated with the practice. Such programmes should equip the traditional slaughter practitioners with the knowledge and skills needed to mitigate both occupational and food-borne risks and thus improve the overall safety of the meat derived from the traditional slaughter of goats.

## Figures and Tables

**Table 1:** Characteristics of the persons involved in the slaughter process.

	Category	Frequency	Percentage
Gender	Male	104	99.00
	Female	1	1.00
Qualifications of slaughterer	Experience	65	62.22
	Designated	37	35.56
	Sangoma	2	2.00
Number involved in slaughter	1–2	43	40.66
	3–4	53	50.55
	5–6	2	2.20
	7>	7	6.59
Knowledge of health status (24 h)	Yes	24	22.47
	No	81	77.53

**Table 2:** Protective clothing worn during the traditional slaughter of goats.

	Category	Frequency	Percentage
Protective clothing	Yes	57	62.64
	No	34	37.36
Change protective clothing after slaughter	Yes	50	58.14
	No	36	41.86
After how long is protective clothing changed?	10 min	26	36.76
	20 min	3	4.41
	30 min	5	7.35
	1 h	5	7.35
	At home	31	44.12
Type of protective clothing worn	Apron	1	1.69
	Overalls	53	93.22
	Own clothes	3	5.08
If no protective clothing, what type of clothes?	Casual clothes	33	97.14
	Sangoma‘s clothes	1	2.86

**Table 3:** Hygiene practices during bleeding and dressing.

	Category	Frequency	Percent
Bleeding time (min)	10–20	78	74.57
	<10	27	25.43
Washing of hands before dressing	Yes	80	87.91
	No	11	12.09
Means of washing hands	Water	49	61.25
	Water and disinfectant	31	38.75
Source of the water	Borehole	8	10.13
	Municipal	69	86.08
	River	3	3.8
Washing of knives before flaying	Yes	77	87.5
	No	11	12.5
Means of washing knives	Water	30	37.97
	Water and disinfectant	45	56.96
	Wet cloth	4	3.8
Frequency of washing knives	After slaughter	2	2.35
	Not at all	11	12.94
	When dirty	72	84.71
Same knife throughout the slaughter	Yes	75	84.27
	No	14	15.73
Other equipment used	Yes	23	25.84
	No	76	74.16
